# The effect of *Ocimum basilicum* on the prevention of postpartum depression symptoms and sleep quality: A triple-blind randomized controlled clinical trial

**DOI:** 10.1016/j.heliyon.2025.e42096

**Published:** 2025-01-20

**Authors:** Monireh Abdi, Elham Rezaei, Mojgan Mirghafourvand, Fatemeh Ebrahimi, Laleh Payahoo, Alireza Shafiei-Kandjani, Solmaz Ghanbari-Homaie

**Affiliations:** a Student Research Committee, Tabriz University of Medical Sciences, Tabriz, Iran; b Department of Midwifery, Faculty of Nursing and Midwifery, Tabriz University of Medical Sciences, Tabriz, Iran; c Department of Traditional Pharmacy, Tabriz University of Medical Sciences, Tabriz, Iran; d Nutrition, Medicinal Plants Research Center, Maragheh University of Medical Sciences, Maragheh, Iran; e Research Center of Psychiatry and Behavioral Sciences, Tabriz University of Medical Sciences, Iran; f Medical Education Research Center, Health Management and Safety Promotion Research Institute, Tabriz University of Medical Sciences, Tabriz, Iran

**Keywords:** Basil, Postpartum depression, Sleep quality, Clinical trial

## Abstract

**Background:**

Postpartum depression is a multifactorial and complex disorder. Various approaches for preventing and treating this condition have been suggested. Nevertheless, there is no definitive proof confirming their efficacy. This study is designed to assess the effectiveness of basil in preventing postpartum depression (primary outcome) and sleep quality (secondary outcome).

**Methods:**

This triple-blind parallel clinical trial (participants, the researcher, and the data analyst were blinded to the assigned interventions) included 78 postpartum women at Taleghani Hospital, Tabriz, Iran, between April 2023 and April 2024. Participants who scored 12 or less on the Edinburgh Postnatal Depression Scale (EPDS) were included in the study. The intervention started within 72 h following birth and continued for eight weeks postpartum. The intervention group received two daily capsules containing dried basil leaf powder, while the control group received 500 mg of starch. The EPDS and postpartum sleep quality scale were completed through structured interviews at eight weeks postpartum. The Mann-Whitney *U* test was used to compare the mean postpartum depression scores before and after the intervention, and ANCOVA was used to compare the mean postpartum sleep quality scores.

**Results:**

The median (25th, 75th percentile) depression score after intervention in the basil and placebo groups was 1.0 (0.0, 2.0) and 1.0 (0.0, 3.0), respectively, with no statistically significant difference between the groups (p = 0.966). The mean (standard deviation) sleep quality score after the intervention in the basil and placebo groups was 17.63 (3.44) and 16.15 (3.20), respectively. There was no statistically significant difference in sleep quality scores between the basil and placebo groups (p = 0.400).

**Conclusion:**

The results show that basil does not prevent postpartum depression or improve sleep quality. Future studies should investigate the effects of basil at higher doses and in extract form.

**Table:** Abbreviation

PPD	Postpartum Depression
DSM-V	Diagnostic and Statistical Manual of Mental Disorders, Fifth Edition
EPDS	Edinburgh Postnatal Depression Scale
SSRI	Selective Serotonin Reuptake Inhibitor
SNRI	Serotonin-Norepinephrine Reuptake Inhibitor
BDNF	Brain-Derived Neurotrophic Factor
NICU	Neonatal Intensive Care Unit
NBF	N-butanol Fraction
EAF	Ethyl Acetate Fraction
WF	Water Fractions
HAE	Hydro Alcoholic Basil Extract

## 1 Introduction

Around 70 % of mothers experience symptoms such as depression, mood fluctuations, and irritability within 2–5 days after giving birth. However, these symptoms do not interfere with their regular activities or involve psychotic symptoms [1,2]. Postpartum blues typically resolve spontaneously after two weeks and should be differentiated from postpartum depression (PPD). However, there is a possibility that some instances may progress into PPD [[3], [4], [5]]. PPD is one of the most common psychological disorders during the first year postpartum [6]. According to the Diagnostic and Statistical Manual of Mental Disorders, Fifth Edition (DSM-V), PPD is defined by the presence of five or more of the following symptoms: 1) depressed mood and significant loss of interest or pleasure; 2) appetite disturbances; 3) sleep disturbances; 4) severe fatigue and loss of energy; 5) feelings of worthlessness or excessive guilt, decreased concentration or decisiveness, and recurrent thoughts of death or suicide [4]. The global prevalence of PPD is estimated to be around 17.7 % [7] and 24 % in Tabriz, Iran [8]. PPD affects a woman's ability to function effectively in many areas of her life [9]. In cases of severe depression, women may experience suicidal, obsessive, and infanticide thoughts [10]. Since postpartum women are also responsible for caring for their newborns, chronic depression can interfere with maternal functioning and result in adverse long-term and short-term outcomes for the child [11,12]. The most significant risk factor for developing PPD is a history of depression during pregnancy. Other important risk factors include low levels of social support throughout life, stressful events during pregnancy, low socioeconomic status, and complications during pregnancy and childbirth [13,14]. Other factors include low education, social isolation, a long interval between marriage and the first pregnancy, marital discord, unintended pregnancy, negative childbirth experience [8], and cesarean birth [15,16].

Another common complaint among postpartum women is poor sleep quality, which can be caused by various factors such as postpartum depression [17,18], the physical, physiological, and psychological effects of childbirth, newborn care [19], and changes in women's roles and social status [20].

Various preventive and therapeutic measures for PPD have been introduced, including psychotherapy [21,22], the prescription of chemical medications such as selective serotonin reuptake inhibitors (SSRIs) [23], and serotonin-norepinephrine reuptake inhibitors (SNRIs) [24]. Complementary and alternative treatment options, such as omega-3 fatty acids [25], exercise [26], massage therapy [27], and acupuncture [28], have also been proposed to reduce the incidence of PPD; however, there is no conclusive evidence regarding their effectiveness. Additionally, studies have shown that medicinal plants such as saffron, turmeric, milk thistle, lavender, ginseng, and rose have antidepressant effects [[29], [30], [31], [32], [33]].

According to studies, basil is one of the plants shown to have antidepressant effects [34]. In animal models, inhalation of basil essential oil has been found to increase serum glucocorticoid levels, reduce neuronal and glial apoptosis, enhance neurogenesis in the dentate gyrus [35], decrease the expression of glucocorticoid receptor and brain-derived neurotrophic factor (BDNF) genes and proteins in the hippocampus, and alleviate depression induced by unpredictable mild stress [36]. In another study, administering basil to male mice with chronic mild stress reduced their depression-like behaviors, corticosterone levels, and hippocampal neuron atrophy and apoptosis [37]. Basil contains significant amounts of linalool, eugenol, and rosmarinic acid [38], all of which have sedative effects [39]. Some animal studies have shown that linalool increases barbiturate-induced sleep time, and eugenol increases sleep duration [40,41]. Rosmarinic acid in basil's methanolic extract also has calming and sleep-inducing effects [42].

There is limited research on basil's impact on depression, especially in humans. No study has been conducted on its effects on reducing the incidence of postpartum depression or its adverse effects on mothers and infants. Given the accessibility and low cost of basil, this study aimed to assess its potential in preventing postpartum depression (the primary objective) and improving sleep quality (the secondary outcome).

## 2 Materials and methods

### 2.1 Study design and data sources

This triple-blind parallel clinical trial (participants, the researcher, and the data analyst were blinded to the assigned interventions) was conducted on 78 postpartum women at Taleghani Teaching, Research, and Treatment Hospital in Tabriz, Iran, between April 2023 and April 2024.

### 2.2 Inclusion and exclusion criteria

The inclusion criteria were term birth and having a healthy newborn. Exclusion criteria included scoring 13 or higher on the Edinburgh Postnatal Depression Scale (EPDS), confirmed mental retardation, confirmed hypertension, a history of confirmed depression, and experiencing a stressful event such as the death or diagnosis of an incurable illness in first-degree family members or divorce within the three months before the study.

### 2.3 Sampling

The researcher (first author) attended the postpartum ward at Taleghani Hospital in Tabriz, Iran, and invited women to participate in the study. If a woman agreed to participate, the eligibility criteria checklist was reviewed, and the EPDS was administered through an interview. Women who scored 12 or less on the EPDS (indicating no depression) were asked to provide written informed consent to participate in the study. Subsequently, sociodemographic and obstetric characteristics questionnaires, as well as the obstetric checklist, were completed through interviews. Each participant was given a package containing 120 capsules to be taken over 60 days (two capsule per day). An individual not involved in sampling, data collection, or analysis prepared the allocation cesequence and packages. The researcher and participants were blinded to the contents of the packages. Participants were orally instructed on how to use the medications and were advised to inform the researcher if any adverse effects occurred.

### 2.4 Sample size

The sample size was calculated based on depression and sleep quality variables using G-Power software. According to the data from the study by Mirghafourvand et al. (2016) [43], regarding the depression variable, with M1 = 8.10, M2 = 5.67 (assuming a 30 % reduction due to intervention), SD1 = SD2 = 3.30, alpha = 0.05 (two-sided), and power = 85 %, the sample size for each group was calculated to be 35. Considering a 10 % dropout rate, the final sample size for each group was calculated to be 39 mothers [43]. Based on the results of the study by Mirghafourvand et al. (2022) [44] regarding the sleep quality variable, with M1 = 22.86, M2 = 16.01 (assuming a 30 % improvement due to intervention), SD1 = SD2 = 8.57, alpha = 0.05 (two-sided), and power = 90 %, the sample size was calculated to be 34 per group. Considering a 10 % dropout rate, the final sample size was 38 mothers per group. Since the sample size calculated based on the depression variable was larger, the final sample size was 39 per group.

### 2.5 Randomization

To minimize bias, the allocation sequence was generated by an individual not involved in the study procedures (corresponding author). Participants were randomly assigned to either the intervention group (basil) or the control group (placebo) using block randomization with blocks of four and six in a 1:1 ratio. The 500 mg basil and starch capsules were placed in consecutively numbered opaque glass bottles without labels, ranging from 1 to 78. The basil and starch capsules were identical in shape and color and packaged in identical bottles. To ensure complete blinding, the starch capsule bottles were scented with basil essential oil. The participants, the researcher, and the outcome analyst were blinded to the randomization status and treatment assignment.

### 2.6 Intervention

The intervention group received two daily capsules containing dried basil leaf powder. In comparison, the control group received two daily capsules (one per 12 h) containing 500 mg of starch. The basil plant, scientifically known as *Ocimum basilicum* L, which belongs to the Lamiacea family, was sourced from a reputable herbal farm in East Azerbaijan province and verified by a at the Pharmacognosy Laboratory of Tabriz Department of Pharmacy. The leaves and aerial parts of the plant were shade-dried and powdered. Under the supervision of a pharmaceutical specialist (fourth author), 500 mg capsules of the plant powder were prepared at the Sina Nowandish Tabiat Co., East Azerbaijan, Tabriz, Iran. Similarly, 500 mg of starch capsules, visually identical to the basil capsules, were prepared for the control group. The intervention began within 72 h post-delivery (cesarean or vaginal) and continued for eight weeks postpartum. The first author administered the Sleep Quality scale in the sixth week and the EPDS in the eighth week through structured interviews with participants. The researcher contacted participants in the first, sixth, and eighth weeks to remind them to take their medications regularly.

### 2.7 Data collection tools

The data collection tools included the sociodemographic characteristics questionnaire, obstetric questionnaire, labor checklist, side effects checklist, Edinburgh Postnatal Depression Scale (EPDS), and postpartum sleep quality scale. All data collection tools in this study were in Persian.

#### 2.7.1 Sociodemographic characteristics questionnaire

This questionnaire recorded the age of the individual and her spouse, their occupations, monthly income, educational levels, and housing status, and was completed at the beginning of the study through a structured interview.

#### 2.7.2 Obstetric checklist

This questionnaire covered gestational age, parity, number of abortions, number of live births and stillbirths, history of prenatal depression, and a history of domestic violence (physical, sexual and emotional types) during pregnancy. It was also completed at the beginning of the study through a structured interview and review of medical record.

#### 2.7.3 Labor checklist

This checklist included details such as the second stage of labor, mode of birth, the feeling of control in pushing during the second stage of labor, shoulder dystocia, Apgar score, degree of tearing, postpartum hemorrhage and prevention methods, NICU admission, use of Remifentanil during labor, use of labor-inducing drugs, and type of infant feeding. The checklist was completed using information recorded in the clinical files with obtaining the participant's permission.*2.7.4. Edinburgh Postnatal Depression Scale (EPDS)*

This questionnaire consists of 10 questions, each rated on a 4-point Likert scale ranging from 0 (low seriousness of the symptom) to 3 (high seriousness of the symptom). The total EPDS score ranges from 0 to 30, with scores of 13 or higher indicating postnatal depression. Questions 1, 2, and 4 are scored from 0 to 3, while questions 3, 5, 6, 7, 8, 9, and 10 are scored from 3 to 0. The psychometric properties of the Persian version of this questionnaire have been evaluated and confirmed in Iran [45,46]. The EPDS was completed twice: once in the first 72 h postpartum (pre-intervention) and again at the end of the intervention at 8 weeks postpartum.

#### 2.7.4 Postpartum sleep quality scale

This scale includes both quantitative aspects of sleep (such as sleep duration and latency) and subjective aspects (such as rest, daily functioning, and factors affecting sleep quality in the postpartum period). It contains 14 questions with five response options (scored from 0 to 4), assessing sleep quality over the past two weeks. The response options are never (0), rarely [1], sometimes [2], often [3], and almost always [4]. Questions 1, 2, and 14 are reversely scored. The total score range is from 0 to 56. Higher scores indicate poorer sleep quality [46]. The psychometric properties of the Persian version have been evaluated and confirmed in Iran [47]. This scale was completed at the end of the intervention, in the eighth week.

#### 2.7.5 Ethical considerations

All stages of this study were conducted following the Helsinki Declaration guidelines. Before the intervention, the researcher explained the study's objectives, methods, and potential side effects to the participants. All participants signed written informed consent forms before participating in the study. The protocol of this study was approved by the Ethics Committee of Tabriz University of Medical Sciences (IR.TBZMED.REC.1401.948) and registered at the Iranian Registry of Clinical Trials (https://irct.behdasht.gov.ir/trial/68333) with the registration number 20220926056046N2.

### 2.8 Data analysis

Data were analyzed using SPSS version 26 (SPSS Inc., Chicago, IL, USA). Each variable's normal distribution was assessed using the Kolmogorov-Smirnov test. The T-test, Chi-square, and Fisher's exact tests were used to compare sociodemographic and obstetric characteristics. Intention to treat analysis was performed. The Mann-Whitney *U* test was used to compare the mean postpartum depression scores before and after the intervention. ANCOVA was used to compare the mean postpartum sleep quality scores. A p-value less than 0.05 was considered statistically significant.

## 3 Results

**Fig. 1:**
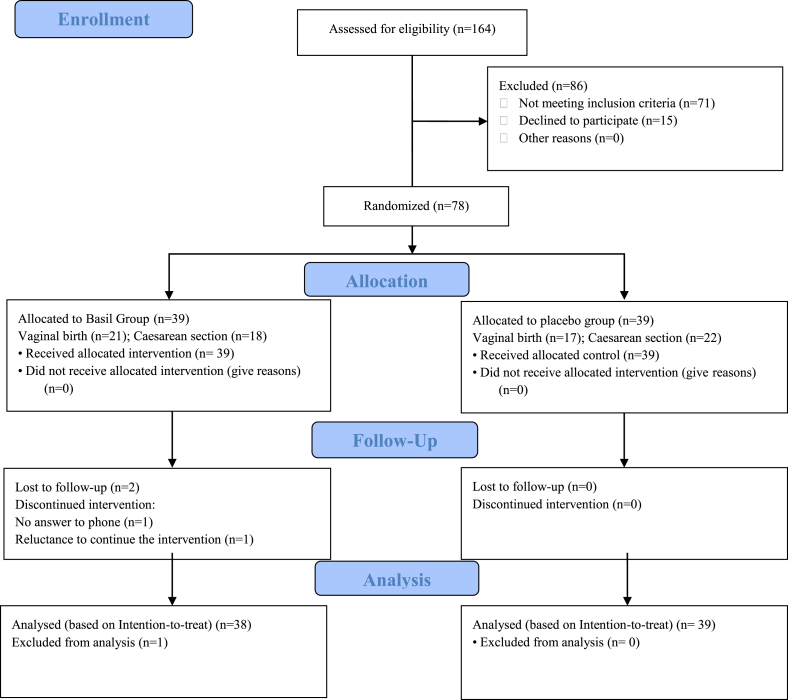
Flow diagram of the study selection process.

Out of 164 postpartum women (cesarean/vaginal birth) assessed, 71 were excluded due to ineligibility and 31 due to unwillingness to participate, leaving 78 eligible women who were recruited for the study. Out of 78 eligible women, 38 and 40 women had cesarean section and vaginal birth, respectively. Then, 39 women were randomly assigned to the intervention group and 39 to the control group. One participant in the intervention group was excluded from the analysis due to non-response to follow-up calls, and another participant who discontinued the intervention due to unwillingness to consume the capsules in the first week, analyzed in the intervention group. Finally, 39 and 38 participants were included in the analysis in the intervention and placebo groups, respectively (Fig. 1).

### 3.1 Sociodemographic and obstetric characteristics outcome

There was no statistically significant difference in all sociodemographic and obstetric characteristics between the intervention and control groups (Table 1, Table 2).

**Table 1:** Demographic characteristics of study participants by group (n = 78).

Variable	Basil Group (n = 39) n (%)	Placebo Group (n = 39) n (%)	p-value
**Age (years)**			0.059^‡^
<25	16 (41.0)	6 (15.4)	
25 to 29	7 [0,18]	6 (15.4)	
30 to 35	6 (15.4)	10 (25.6)	
>35	10 (25.6)	17 (43.6)	
**Husband's Age (years)**			0.156^‡^
<25	3 (7.7)	2 (5.1)	
25 to 29	12 (30.8)	6 (15.4)	
30 to 35	9 (23.0)	6 (15.4)	
>35	15 (38.5)	25 (64.1)	
**Education Level**			0.061^§^
Primary	6 (15.4)	14 (35.9)	
Middle School	27 (69.2)	17 (43.5)	
High School	5 (12.8)	4 (10.3)	
University	1 (2.6)	4 (10.3)	
**Husband's Education Level**			0.223^§^
Primary	3 (7.7)	6 (15.4)	
Middle School	28 (71.8)	20 (51.3)	
High School	7 (17.9)	9 (23.0)	
University	1 (2.6)	4 (10.3)	
**Employment Status**			1.000^‡^
Housewife	38 (97.4)	38 (97.4)	
Employed	1 (2.6)	1 (2.6)	
**Husband's Employment Status**			0.176^‡^
Self-employed	36 (92.3)	32 (82.1)	
Employed	3 (7.7)	7 (17.9)	
**Housing Status**			0.808^‡^
Personal	26 (66.7)	27 (69.2)	
Rental	13 (33.3)	12 (30.8)	
**Satisfaction of Income Level**			0.200^§^
Completely Satisfied	1 (2.6)	4 (10.3)	
Completely Dissatisfied	0 (0)	1 (2.6)	
Relatively Satisfied	38 (97.4)	34 (87.1)	
**Domestic Violence**			1.000^§^
Yes	2 (5.1)	0 (0.0)	
No	37 (94.9)	39 (100)	

∗Independent *t*-test.

^‡^Fisher's exact test.

^§^Chi-square test.

**Table 2:** Obstetric characteristics of study participants by group (n = 78).

Variable	Basil Group (n = 39) n (%)	Placebo Group (n = 39) n (%)	p-value
**Gestational Age (weeks), Mean (SD)**	38.3 (1.0)	38.3 (1.0)	0.834^a^
**Number of Pregnancies**			0.467^c^
1	15 (38.5)	10 (25.6)	
2	14 (35.9)	14 (35.9)	
3	10 (25.6)	15 (38.5)	
**Parity**			0.400^c^
1	15 (38.5)	10 (25.6)	
2	14 (35.9)	14 (35.9)	
3	10 (25.6)	15 (38.5)	
**Number of Live Children**			0.607^c^
1	14 (35.9)	11 (28.2)	
2	15 (38.5)	14 (35.9)	
3	10 (25.6)	14 (35.9)	
**History of Abortion**			0.769^c^
0	31 (79.5)	33 (84.6)	
1	8 (20.5)	6 (15.4)	
**History of Stillbirths**			0.464^b^
No	37 (94.9)	39 (100)	
Yes	2 (5.1)	0 (0.0)	
**Unwanted Pregnancy**			1.000^b^
Yes	1 (2.6)	0 (0.0)	
No	37 (97.4)	39 (100)	
**History of Infertility**			0.615^b^
Yes	2 (2.6)	3 (7.7)	
No	37 (97.4)	36 (92.3)	
**Second Stage of Labor (min)**			0.136^b^
30	9 (42.9)	11 (64.7)	
50	9 (42.9)	6 (35.3)	
60	3 (14.2)	0 (0.0)	
**The Feeling of Control During vaginal Birth**			0.427^b^
Yes	16 (76.2)	15 (88.2)	
No	5 (23.8)	2 (11.8)	
**Mode of Birth**			0.497^c^
Vaginal	21 (53.8)	17 (43.6)	
Cesarean	18 (46.2)	22 (56.4)	
**Postpartum Hemorrhage Control**			0.238^c^
Oxytocin	28 (71.8)	22 (56.4)	
Misoprostol and Oxytocin	11 (28.2)	17 (43.6)	
**Augmentation of labor in Vaginal Birth**			0.318^c^
Yes	15 (71.4)	9 (52.0)	
No	6 (28.6)	8 (47.0)	
**Apgar Score (1**st **Minute)**			0.711^c^
Score 8	3 (7.7)	5 (12.8)	
Score 9	36 (92.3)	34 (87.2)	
**Apgar Score (5th Minute)**			0.263^b^
Score 9	2 (5.1)	6 (15.4)	
Score 10	37 (94.9)	33 (84.6)	
**NICU Admission**			0.755^b^
Yes	5 (12.8)	7 (17.9)	
No	34 (87.2)	32 (82.1)	
**Maternal Immobility During First Stage of Labor**			0.307^c^
Yes	5 (23.8)	7 (41.2)	
No	16 (76.2)	10 (58.8)	
**Use of Remifentanil**			0.502^c^
Yes	15 (71.4)	9 (53.0)	
No	6 (28.6)	8 (47.0)	
**Infant Feeding in the First Six Weeks**			0.911^b^
Breast Milk	29 (74.4)	31 (79.5)	
Formula	2 (5.1)	2 (5.1)	
Breast Milk and Formula	8 (20.5)	6 (15.4)	

a Independent *t*-test.

b Fisher's exact test.

c Chi-square test.

### 3.2 Postpartum depression before intervention outcome

The median (25th, 75th percentile) postpartum depression score before intervention in the basil and placebo groups was 0.1 (0.0, 3.0) and 0.1 (0.0, 3.0), respectively, with no statistically significant difference between the two groups (p = 0.353).

### 3.3 Postpartum depression after intervention outcome

Additionally, the median (25th, 75th percentile) postpartum depression score after the intervention in the basil and placebo groups was 1.0 (0.0, 2.0) and 1.0 (0.0, 3.0), respectively, with no statistically significant difference between the basil and placebo groups (p = 0.966) (Table 3).

**Table 3:** Comparison of overall depression scores between study groups.

Variable	Basil Group Before Intervention (n = 39) After Intervention (n = 38)^b^		Placebo Group (n = 39)		p-value
	Median (25th, 75th Percentile)	Mean (SD)	Median (25th, 75th Percentile)	Mean (SD)	
**Depression Before Intervention**	1.0 (0.0, 3.0)	1.66 (1.79)	1.0 (0.0, 3.0)	2.28 (2.32)	0.353^a^
**Depression After Intervention**	1.0 (0.0, 2.0)	1.63 (2.22)	1.0 (0.0, 3.0)	1.61 (2.14)	0.968^a^

a Mann-Whitney *U* Test.

b One participant excluded from analysis.

### 3.4 Sleep quality after intervention outcome

The mean (standard deviation) sleep quality score after the intervention in the basil and placebo groups was 17.63 (3.44) and 16.15 (3.20), respectively. According to the ANCOVA test, there was no statistically significant difference in sleep quality scores between the basil and placebo groups (p = 0.400) (Table 4).

**Table 4:** Comparison of mean overall sleep quality scores among postpartum women by study group.

Variable	Basil Group b(n = 38) Mean (SD)	Placebo Group (n = 39) Mean (SD)	Mean Difference (95 % CI)	p-value
**Sleep Quality After Intervention**	17.63 (3.44)	16.15 (3.20)	1.7 (−0.01, 3.2)	0.400^a^

a ANCOVA Test Adjusted for Baseline Values.

b One participant excluded from analysis.

### 3.5 Participant satisfaction

In the basil-receiving group, 35 participants (94.6 %) were very satisfied or satisfied. In contrast, in the control group, 37 participants (94.8 %) were very satisfied or satisfied. Fisher's exact test indicated no statistically significant difference between the two groups in terms of satisfaction with the drug (p = 1.000). Also, 35 participants in the basil group (94.6 %) and 37 participants in the control group (94.9 %) were satisfied or satisfied with the received capsules. There was no statistically significant difference between the groups in terms of treatment response from the participants' perspective (p = 0.456) (Table 5).

**Table 5:** Participant satisfaction and side effects by study group.

Variable	bBasil Group (n = 37) n (%)	Placebo Group (n = 39) n (%)	p-value
**Satisfaction**			0.456^a^
Very satisfied	10 (27.0)	6 (15.4)	
Satisfied	25 (67.6)	31 (79.5)	
Dissatisfied	2 (5.4)	2 (5.1)	
**Side Effects**			1.000^a^
Yes	0.0	1 (2.6)	
No	37 (100)	38 (97.4)	

a Fisher's Exact Test.

b Two participant excluded from analysis.

### 3.6 Side effects

Regarding side effects, one participant in the intervention group reported nausea after taking the capsules in the third week (Table 5).

## 4 Discussion

The present study indicated that the daily consumption of two 500-mg basil capsules for eight weeks does not affect the prevention of postpartum depression. To our knowledge, this is the first study to measure the effect of basil on postpartum depression. Most studies in this area have investigated animal cases. Our study results are not consistent with some studies that have examined the effect of basil and its relatives on depression and stress. A triple-blind, controlled clinical trial by Karimi et al. assessed the impact of oral capsules of basil leaf extract on depression in menopausal women. Menopausal women received one 500-mg basil leaf extract capsule or placebo daily for one month. According to the findings, after the intervention, the mean depression score in the basil group was significantly lower than in the placebo group [34].

One of the reasons for the inconsistency of two studies may be the amount of active ingredients of the capsules. In our study the capsules were filled with plant powder whereas in karimi's study the plant extract was used to formulate the capsules. Obviously, the concentration of active ingredients effective on depression and sleep disorders (mainly: linalool, eugenol and rosmarinic acid) in extract is higher in comparison to the plant powder. Furthermore, the content and composition of active substances in medicinal plants is significantly influenced by various ecological factors such as: cultivation areas, climatic variables, soil characteristics and biological factors [48]. In this study, the basil plant was obtained from East Azerbaijan Province. Its aerial parts were dried and used as powder in 500-mg capsules; In a study conducted by Alex N Yosif et al., air-drying basil was associated with a reduction in volatile compounds, including linalool, while in Karimi et al.'s study, the basil plant was obtained from a farm affiliated with Ferdowsi University of Mashhad. The aerial parts were powdered and soaked in a 70 % hydroalcoholic solution for 3–5 days. The hydroalcoholic extract was combined with Avicel and used as a powder in 500-mg capsules Another reason for the inconsistency may be this point that the nature of postpartum depression is different from depression caused by other factors, and postpartum depression can be considered a distinct disorder [49,50]. Postpartum depression is a complex disorder, but its exact mechanism and all related factors have not been identified. Therefore, various genetic, biological, and environmental factors can expose women to depression after childbirth. Furthermore, many physical, psychological, hormonal, and other stressors can exacerbate this disorder during pregnancy. Thus, the complex and multifactorial nature of postpartum depression may be one of the reasons for not observing a significant difference between the two groups in the present study [51]. Another reason for the mentioned study's inconsistency could be the differing objectives of the two investigations. The present study aimed to prevent depression, while Karimi et al. were to treat depression. Additionally, given that the prevalence of depression among Iranian women is approximately 11 % [52] and that depression was evaluated eight weeks after the intervention, it is feasible that the results would differ if the intervention were to persist and depression were assessed in subsequent months. Another reason for not detection the effect may be that women did not report symptoms due to feelings of shame and fear of being judged and avoiding the label of mental illness [53,54]. An animal study examined basil's analgesic and sedative effects on 20 male rats. In this study, alcoholic basil extracts were used at doses of 50 and 100 mg per kilogram. The results showed that 100 mg of basil extract significantly decreased pain response. Additionally, the 100-mg dose had a strong sedative effect. This study reported the dose-dependent impact of basil [55]. A controlled triple-blind clinical trial reported that lemon balm (Melissa Officinalis) (from the Mint family) reduced postpartum depression symptoms in women undergoing cesarean sections [56].

A systematic review examining the effect of herbal remedies, including lavender (from the mint family), on postpartum depression reported the positive impact of lavender on postpartum depression and sleep disorders [57]. The current study showed that the daily consumption of two 500 mg basil capsules for eight weeks did not significantly improve postpartum sleep quality. We couldn't find any studies investigating the effect of basil on sleep quality in humans. The long-term effects of hydroalcoholic basil extract (HAE) on male mice's sleep were examined to determine if HAE has a sleep-prolonging effect. Animals in the control group received saline and diazepam (as a positive control), and in the intervention group, different doses of HAE (25, 50, and 100 mg) were administered. In the second experiment, to determine the most effective part of HAE, animals were treated with N-butanol (NBF), ethyl acetate (EAF), and water fractions (WF), all obtained from the distillation of basil extract. According to the results of this study, all three doses had a positive effect on prolonging sleep, and among the compounds, NBF induced the maximum prolongation of sleep [58].

### 4.1 Strengths and limitations

The strengths of this study include random allocation, allocation concealment, and a triple-blind design. One limitation of the current study was the lack of investigation and control of sleep disruptors such as maternal sleep status during the pre-pregnancy and postpartum periods, type of breastfeeding (breast milk or formula) [59]. and infant's colic and jaundice status [60], which can affect the mother's sleep quality. One limitation of this study is the lack of a long-term evaluation of depression symptoms and the short intervention period. Also, the current research is limited in that it does not assess and control factors such as maternal childbirth experience [8] and social support [61,62] which influence the occurrence of depression. Since our diagnostic method for postpartum depression was based on the questionnaire rather than a diagnosis by a psychologist or psychiatrist, this could be one of the limitations in our study. It is recommended that future studies use different doses and forms of basil preparations. It is also suggested that the effect of this plant be evaluated among women suffering from postpartum depression.

## 5 Conclusion

The current study indicates that basil does not prevent postpartum depression or improve sleep quality. Future studies should investigate the effect of basil at higher doses and in extract form.

## CRediT authorship contribution statement

**Monireh Abdi:** Writing – review & editing, Investigation, Conceptualization. **Elham Rezaei:** Writing – original draft. **Mojgan Mirghafourvand:** Writing – review & editing, Formal analysis. **Fatemeh Ebrahimi:** Writing – review & editing, Investigation. **Laleh Payahoo:** Writing – review & editing, Conceptualization. **Alireza Shafiei-Kandjani:** Writing – review & editing, Validation. **Solmaz Ghanbari-Homaie:** Writing – review & editing, Conceptualization.

## Data availability

The datasets generated and/or analyzed during the current study are not publicly available due to ethical restrictions regarding patient data and anonymity. Still, they are available from the corresponding author upon reasonable request.

## Ethical considerations

All stages of this study, conducted on human samples, followed the Helsinki Declaration guidelines and regulations. The Ethics Committee of Tabriz University of Medical Sciences, Tabriz, Iran (IR.TBZMED.REC.1401.948) approved the study protocol and the registration date in IRCT was prior to the sampling (2023.2.21). All participants gave informed written consent and were fully informed of the research objectives and methods. Anonymity was preserved through questionnaire coding.

## Funding

This paper is the result of a master's thesis of first author. This study was supported and funded by 10.13039/501100004366Tabriz University of Medical Sciences, Iran (Code: 70892).

## Declaration of competing interest

The authors declare that they have no known competing financial interests or personal relationships that could have appeared to influence the work reported in this paper.
